# *TERT* promoter mutations and recurrence patterns in differentiated thyroid carcinoma

**DOI:** 10.1530/ERC-25-0273

**Published:** 2026-02-16

**Authors:** Hyun Jin Ryu, Ji Hyun Yoo, Da Eun Leem, Chang-Seok Ki, Jung Hee Shin, Young Lyun Oh, Jung-Han Kim, Ji Eun Jun, Kyu Jeung Ahn, Joonghyun Ahn, Sun Wook Kim, Jae Hoon Chung, Tae Hyuk Kim, Young Ik Son

**Affiliations:** ^1^ Division of Endocrinology and Metabolism, Department of Internal Medicine, Kyung Hee University School of Medicine, Kyung Hee University Hospital at Gangdong, Seoul, Korea; ^2^ Division of Endocrinology and Metabolism, Department of Medicine, Thyroid Center, Samsung Medical Center, Sungkyunkwan University School of Medicine, Seoul, Korea; ^3^ Department of Internal Medicine, Korea Cancer Center Hospital, Seoul, Korea; ^4^ Green Cross Genome, Yongin, Korea; ^5^ Department of Radiology, Samsung Medical Center, Sungkyunkwan University School of Medicine, Seoul, Korea; ^6^ Department of Pathology and Translational Genomics, Samsung Medical Center, Sungkyunkwan University School of Medicine, Seoul, Korea; ^7^ Division of Breast and Endocrine Surgery, Department of Surgery, Samsung Medical Center, Sungkyunkwan University School of Medicine, Seoul, Korea; ^8^ Statistic and Data Center, Clinical Research Institute, Samsung Medical Center, Seoul, Korea; ^9^ Department of Otorhinolaryngology-Head and Neck Surgery, Samsung Medical Center, Sungkyunkwan University School of Medicine, Seoul, Korea

**Keywords:** *TERT* promoter mutations, differentiated thyroid carcinoma, recurrence, prognosis

## Abstract

Telomerase reverse transcriptase promoter mutations (*TERT*) are known prognostic factors associated with poor outcomes in differentiated thyroid carcinoma (DTC). We analyzed differences in DTC recurrence patterns over time according to *TERT*. A retrospective review was conducted on DTC patients who achieved remission after total thyroidectomy and/or radioactive iodine treatment at Samsung Medical Center between 1994 and 2004. The sites and patterns by time of recurrence in the patients were reviewed. In total, 367 patients with a median follow-up of 14 years (interquartile range, 12–17 years) were included. Recurrence occurred in 91 patients, wherein 56 had lymph node recurrence and 35 had either local or distant recurrence. *TE* *RT* and tumor size <2 cm were independent factors for recurrence in the Cox proportional hazards analysis. In cases of *TERT*-wild type (WT), the recurrence rate decreased significantly over time after diagnosis, whereas in *TERT*-mutant type (MT), a consistently high recurrence rate was observed. In the Kaplan–Meier analysis, *TERT*-MT exhibited a significantly poorer disease-free survival, with continuous recurrence over time, whereas in *TERT*-WT, the curve showed a gradual decline. Further analysis of the overall survival among the 91 patients revealed that *TERT*-MT was significantly associated with a higher risk of mortality, whereas *TERT*-WT exhibited a slowly decreasing curve. In conclusion, *TERT*-MT was a significant adverse prognostic factor wherein continuous recurrence necessitates long-term follow-up. For *TERT*-WT, the recurrence rate decreased compared to *TERT*-MT, and even in cases of recurrence, the treatment outcomes are favorable.

## Introduction

Differentiated thyroid cancer (DTC), predominantly papillary thyroid cancer (PTC) or follicular thyroid cancer (FTC), comprises over 90% of thyroid cancer cases and generally demonstrates an indolent prognosis (1, 2, 3). The 2015 American Thyroid Association (ATA) recommended a less extensive therapy for DTC management (4). However, some studies have demonstrated an increase in the rates of advanced-stage disease and incidence-based mortality rates and that approximately 15–20% of PTC patients have recurrences or a progressive disease course (5, 6).

Telomerase reverse transcriptase promoter mutations (*TERT*) are well-known adverse prognostic factors for DTC recurrence and mortality (7, 8, 9). Many studies have proposed the use of *TERT* to stratify risk and subsequently guide treatment in DTC patients with long survival (10, 11, 12).

Hence, this study analyzed the impact of *TERT* on recurrence, specifically stratified by recurrence location. Considering that PTC constitutes a significant proportion of DTC cases, and that recurrences in DTC primarily occur as lymph node recurrence (LNR) patterns (13, 14), this study classified the recurrence type as LNR vs local soft tissue recurrence (LR) or distant recurrence (DR; LR/DR). By focusing on recurrence patterns, this study aimed to explore whether the prognostic impact of *TERT* differs by recurrence type, beyond the general association with recurrence or mortality.

## Methods

### Selection of patients and their clinicopathologic data

A retrospective review was conducted on patients who were treated for DTC and achieved remission at Samsung Medical Center between 1994 and 2004. Patients with distant metastases at diagnosis or those lost to follow-up during treatment were excluded. This study was conducted according to the guidelines of the 1964 Declaration of Helsinki. This study was approved by the Institutional Review Board of the Samsung Medical Center (SMC-IRB 2016-05-053), and the requirement for written informed consent was waived because of the retrospective nature of this study.

The definitions of remission and recurrence were consistent with those in a previous study (15). Remission was defined as stimulated serum thyroglobulin (Tg) <2 ng/mL with undetectable anti-Tg antibodies (<60 IU/mL), a significant absence of abnormal uptake on a diagnostic whole-body scan in the neck region, and negative findings on ultrasonography after the initial therapy. If a recurrent lesion was detected following remission, it was documented as a recurrence. LNR incorporated recurrences in either the central or lateral lymph nodes. LR was defined as a recurrence in the thyroid bed or perithyroidal soft tissue, excluding the lymph nodes. Diagnoses of both LNR and LR were achieved through pathological confirmation following aspiration or reoperation. DR was detected through pathological confirmation or imaging examinations, such as whole-body scan, computed tomography (CT), magnetic resonance imaging (MRI), or positron emission tomography (PET) (4).

Various factors that could affect DTC recurrence were incorporated into the analysis as described previously (15). Age at the initial diagnosis and sex were included as patient factors. The type of surgery (total or near-/subtotal thyroidectomy), radioactive iodine (RAI) therapy, and initial central neck dissection were included as treatment factors. *TERT*, tumor subtype (PTC or FTC), histology (favorable vs unfavorable), tumor size, multifocality, extrathyroidal extension, resection margins, and lymph node metastasis were included as tumor factors. Multifocality was defined as the presence of two or more distinct areas of DTC within the surgical specimen. Unfavorable tumor histology includes tall cell, diffuse sclerosing, columnar cell, solid, and hobnail variants of PTC (4) and the widely invasive follicular thyroid carcinoma of FTC (16).

Prophylactic central neck dissection (ipsilateral or bilateral) was performed in 63.5% of patients. Therapeutic lateral neck dissection was performed in patients with biopsy-confirmed lateral cervical lymph node metastases. Most patients (92.6%) received RAI after levothyroxine withdrawal or recombinant human thyrotropin stimulation. An initial dose of 30–200 mCi ^131^I was administered for remnant ablation or treatment of residual or metastatic disease.

Posttreatment monitoring and management were performed as previously described (15). Serum Tg and anti-Tg antibody levels were measured at 3 months after surgery, every 6 months for the first 5 years, and every 12 months thereafter. Following surgery, cervical ultrasound was performed at 6–12 months and then periodically depending on the disease risk and Tg status of the patient. Various imaging modalities, including neck and chest CT, MRI, and PET scans, and cytological/histological proof were used to detect recurrent or metastatic lesions. Thyroid-stimulating hormone (TSH) suppression therapy with levothyroxine was continued to attain optimal serum TSH levels.

All patients were initially staged according to the American Joint Committee on Cancer tumor/node/metastasis (TNM) classification system (eighth edition).

### Detection of *TERT*

Genomic DNA was extracted from all available tissue blocks using a Qiagen DNA formalin-fixed, paraffin-embedded tissue kit (Qiagen, Germany) according to the manufacturer’s instructions. Promoter mutations in the *TERT* gene were ascertained by semi-nested polymerase chain reaction and sequencing as previously described (10).

### Statistical analysis

Continuous data were expressed as mean ± standard deviation (SD) or median (range, min–max). Categorical characteristics are expressed as absolute numbers (percentages). For the comparison of clinical and pathological characteristics among the groups, Fisher’s exact test was used for categorical data and the *t*-test was used for continuous data. Disease-free survival (DFS) and overall survival (OS) were summarized using Kaplan–Meier curves, and comparisons between patient groups were made using the log-rank test. For DFS, the baseline time point was the date of thyroid cancer surgery, and DFS was measured from surgery to the first recurrence. For OS, the baseline time point was the date of the first recurrence, and OS was measured from first recurrence to death or last contact. The effect of *TERT* on overall prognosis has already been established, and we therefore analyzed whether it also holds significance in this high-risk subgroup of patients who had already experienced recurrence. A multivariate Cox proportional hazards model was used to evaluate the association between *TERT* and time-to-event outcomes. The primary aim of this analysis was to adjust for potential confounders in assessing the relationship between *TERT* and outcomes. Candidate variables for multivariable Cox regression were prespecified based on clinical relevance. To avoid univariable screening and to account for potential correlations among covariates, a forward stepwise selection procedure was applied to derive the final multivariable models. The proportional hazard assumption was assessed using the Schoenfeld residual test. Statistical significance was set at *P* < 0.05. All statistical analyses were performed using R, version 4.3.0 (R Foundation for Statistical Computing, Austria), and SAS, version 9.4 (SAS Institute Inc., USA).

## Results

### Clinicopathological characteristics of the recurrence and NED groups

The clinicopathological characteristics of the 367 patients who achieved remission after optimal DTC therapy with a follow-up duration of 14 ± 3.13 years are shown in Table 1. The recurrence and NED groups were compared. Among the 91 patients with total recurrence (TR), 56 experienced LNR and 35 experienced LR/DR. Postoperatively, 340 (92.6%) patients underwent RAI, with a higher proportion of patients in the TR group receiving RAI treatment (no recurrence vs TR, 250/276 (90.6%) vs 90/91 (98.9%), *P* = 0.005). *TERT* were detected in 31 patients (8.4%), and its prevalence was significantly higher in the TR group than in the non-recurrence group (no recurrence vs TR, 10/276 (3.6%) vs 21/91 (23.1%), *P* < 0.001). However, no significant difference was observed between the types of recurrence (LNR vs LR/DR, 13/56 (23.2%) vs 8/35 (22.9%), *P* = 1.000). The proportion of patients with PTC was significantly higher in the TR group than in the non-recurrence group (no recurrence vs TR, 227/276 (82.2%) vs 84/91 (92.3%), *P* = 0.019). Among recurrences, there was a relatively higher proportion of PTC in the LNR group than in the LR/DR group (LNR vs LR/DR, 54/56 (96.4%) vs 30/35 (85.7%), *P* = 0.144). The median primary tumor size was 2.7 cm (range, 0.4–12.0 cm), and it was significantly smaller in the recurrence group compared to the non-recurrence group (no recurrence vs TR, median 2.8 cm (range, 0.4–12.0 cm) vs 2.5 cm (range, 0.4–10.5 cm), *P* = 0.047). There was a significantly higher prevalence of positive resection margins in the TR group than in the non-recurrence group (no recurrence vs TR, 35/276 (13.5%) vs 24/91 (26.7%), *P* = 0.007). There was a significantly higher prevalence of lateral lymph node metastasis in the TR group than in the non-recurrence group (no recurrence vs TR, 39/276 (14.1%) vs 26/91 (28.5%), *P* = 0.005). The proportion of patients with stage I disease was lower in the TR group than in the non-recurrence group (no recurrence vs TR, 256/276 (92.8%) vs 72/91 (79.1%), *P* = 0.001), and a higher proportion of patients with stage II disease was observed in the TR group (no recurrence vs TR, 15/276 (5.4%) vs 16/91 (17.6%), *P* = 0.001).

**Table 1:** Characteristics of patients according to the recurrence patterns.

Characteristics	Total (*n* = 367)	No recurrence (*n* = 276)	Recurrence pattern			*P*
			TR (*n* = 91)	LNR (*n* = 56)	LR/DR (*n* = 35)	
Patient factors						
Age	42.3 (15.0–81.4)	41.8 (15.8–76.1)	43.8 (15.8–81.4)	41.7 (15.8–81.4)	47.2 (20.0–75.1)	0.125
Age < 55	303 (82.6)	239 (86.6)	64 (70.3)	41 (73.2)	23 (65.7)	
Female	309 (84.2)	234 (84.8)	75 (82.4)	48 (85.7)	27 (77.1)	0.620
Treatment factors						
Type of thyroid surgery (total)	345 (94.0)	258 (93.5)	87 (95.6)	55 (98.2)	32 (91.4)	0.726
RAI	340 (92.6)	250 (90.6)	90 (98.9)	56 (100.0)	34 (97.1)	0.005
Initial CND	233 (63.5)	172 (62.3)	61 (67.0)	41 (73.2)	20 (57.1)	0.453
Tumor factors						
*TERT* (MT)	31 (8.4)	10 (3.6)	21 (23.1)	13 (23.2)	8 (22.9)	<0.001
Subtypes (PTC)	311 (84.7)	227 (82.2)	84 (92.3)	54 (96.4)	30 (85.7)	0.019
Histology (favorable)	341 (93.7)	254 (93.0)	87 (95.6)	54 (96.4)	33 (94.3)	0.465
Tumor size (cm)	2.7 (0.4–12.0)	2.8 (0.4–12.0)	2.5 (0.4–10.5)	2.2 (0.5–5.0)	2.8 (0.4–10.5)	0.047
<2 cm	40 (10.9)	17 (6.2)	23 (25.3)	13 (23.2)	10 (28.6)	
2–4 cm	280 (76.3)	221 (80.1)	59 (64.8)	40 (71.4)	19 (54.3)	
>4 cm	47 (12.8)	38 (13.8)	9 (9.9)	3 (5.4)	6 (17.1)	
Multifocal tumor	98 (26.7)	70 (25.4)	28 (30.8)	21 (37.5)	7 (20.0)	0.340
Gross ETE						0.678
Negative	334 (91.0)	251 (90.9)	83 (91.2)	50 (89.3)	33 (94.3)	
Only strap muscle	21 (5.7)	17 (6.2)	4 (4.4)	4 (7.1)	0 (0.0)	
Major neck structures	12 (3.3)	8 (2.9)	4 (4.4)	2 (3.6)	2 (5.7)	
Resection margin	59 (16.9)	35 (13.5)	24 (26.7)	14 (25.5)	10 (28.6)	0.005
Central LNM	153 (41.7)	109 (39.5)	44 (48.4)	31 (55.4)	13 (37.1)	0.143
Lateral LNM	65 (17.7)	39 (14.1)	26 (28.6)	17 (30.4)	9 (25.7)	0.003
TNM stage						0.001
Stage I	328 (89.4)	256 (92.8)	72 (79.1)	45 (80.4)	27 (77.1)	
Stage II	31 (8.4)	15 (5.4)	16 (17.6)	10 (17.9)	6 (17.1)	
Stage III	8 (2.2)	5 (1.8)	3 (3.3)	1 (1.8)	2 (5.7)	

The *P*-value was computed to compare the no recurrence group with the TR group. The values are presented as the median (minimum value–maximum value) or number (percentage).

TR, total recurrence; LNR, lymph node recurrence; LR/DR, local soft tissue recurrence or distant recurrence; RAI, radioactive iodine therapy; CND, central neck dissection; *TERT*, telomerase reverse transcriptase; MT, mutant type; PTC, papillary thyroid carcinoma; ETE, extrathyroidal extension; LNM, lymph node metastasis; and TNM, tumor/node/metastasis.

Additional baseline characteristics stratified by *TERT* mutational status (WT vs MT) are summarized in Supplementary Table 1 (see section on Supplementary materials given at the end of the article).

### Risk factors for recurrence

Independent factors for DTC recurrence were examined using Cox regression analysis (Table 2). Using univariate Cox regression analysis, age ≥55 years, *TERT*-mutant type (MT), PTC subtype, size ≥2 cm, positive resection margin, and lateral LNM were associated with TR. A Cox proportional hazard regression model incorporating these variables revealed that *TERT*-MT (hazard ratio (HR): 2.813, 95% confidence interval (CI): 1.513–5.230), size ≥2 cm (2–4 cm, HR: 0.184, 95% CI: 0.110–0.308; >4 cm, HR: 0.255, 95% CI: 0.114–0.571), and positive resection margin (HR: 1.885, 95% CI: 1.135–3.129) were significant predictors for TR.

**Table 2:** HR of *TERT* promoter mutations for recurrence according to the location.

Variable (reference)	TR				LNR				LR/DR			
	Unadjusted		Adjusted		Unadjusted		Adjusted		Unadjusted		Adjusted	
	HR (95%CI)	*P*	HR (95%CI)	*P*	HR (95%CI)	*P*	HR (95%CI)	*P*	HR (95%CI)	*P*	HR (95%CI)	*P*
Age (<55)	2.460 (1.566–3.865)	<0.001	1.650 (0.946–2.876)	0.077	1.900 (1.080–3.344)	0.026			3.051 (1.509–6.170)	0.002	2.649 (1.187–5.913)	0.017
Sex (female)	1.257 (0.732–2.159)	0.406			1.193 (0.624–2.282)	0.594			1.765 (0.799–3.900)	0.160	2.074 (0.892–4.823)	0.090
Type of surgery (total)	0.744 (0.374–1.479)	0.399			0.253 (0.042–1.520)	0.133			1.237 (0.582–2.631)	0.581		
RAI (not done)	7.991 (1.113–57.360)	0.039			-	-			2.874 (0.393–21.020)	0.298		
Initial CND (not done)	1.169 (0.755–1.810)	0.485			1.401 (0.821–2.392)	0.216			0.752 (0.385–1.470)	0.405		
*TERT* (WT)	4.466 (2.730–7.306)	<0.001	2.813 (1.513–5.230	0.001	4.300 (2.373–7.793)	<0.001	4.785 (2.602–8.799)	<0.001	4.072 (1.830–9.062)	0.001	2.466 (0.989–6.149)	0.053
Subtype (PTC)	0.434 (0.201–0.938)	0.034	0.495 (0.220–1.111)	0.088	0.253 (0.079–0.806)	0.020	0.302 (0.090–1.022)	0.054	0.869 (0.337–2.243)	0.772		
Histology (favorable)	0.682 (0.250–1.858)	0.454			0.462 (0.113–1.890)	0.283			0.934 (0.224–3.899)	0.926		
Tumor size												
<2 cm	1.0 (reference)	-			1.0 (reference)	-			1.0 (reference)	-		
2–4 cm	0.256 (0.157–0.417)	<0.001	0.184 (0.110–0.308)	<0.001	0.267 (0.150–0.476)	<0.001	0.206 (0.114–0.375)	<0.001	0.191 (0.088–0.417)	<0.001	0.142 (0.063–0.323)	<0.001
>4 cm	0.261 (0.120–0.566)	<0.001	0.255 (0.114–0.571)	<0.001	0.200 (0.073–0.548)	0.002	0.243 (0.088–0.675)	0.007	0.415 (0.150–1.150)	0.091	0.406 (0.141–1.170)	0.094
Multifocal tumor (negative)	1.274 (0.816–1.988)	0.287			1.689 (1.020–2.797)	0.042	1.569 (0.932–2.641)	0.090	0.707 (0.309–1.618)	0.411		
Gross ETE (negative)	1.143 (0.704–1.855)	0.589			1.107 (0.613–1.999)	0.736			0.991 (0.424–2.314)	0.983		
Resection margin (negative)	1.980 (1.240–3.164)	0.004	1.885 (1.135–3.129)	0.014	1.656 (0.928–2.954)	0.088			2.205 (1.054–4.612)	0.036	2.931 (1.322–6.499)	0.008
Central LNM (negative)	1.343 (0.890–2.027)	0.160			1.568 (0.963–2.552)	0.070	1.496 (0.897–2.494)	0.123	0.848 (0.427–1.685)	0.638		
Lateral LNM (negative)	2.221 (1.407–3.505)	<0.001	1.569 (0.921–2.671)	0.097	1.958 (1.125–3.407)	0.018			1.921 (0.896–4.117)	0.093		

HR, hazard ratio; *TERT*, telomerase reverse transcriptase; TR, total recurrence; LNR, lymph node recurrence; LR/DR, local soft tissue recurrence or distant recurrence; CI, confidence interval; RAI, radioactive iodine therapy; CND, central neck dissection; WT, wild type; PTC, papillary thyroid carcinoma; ETE, extrathyroidal extension; and LNM, lymph node metastasis.

When analyzing recurrence in terms of LNR and LR/DR, the factors associated with LNR in the univariate survival analysis included age ≥55 years, *TERT*-MT, PTC subtype, size ≥2 cm, multifocality, and lateral LNM. Among the variables, *TERT*-MT (HR: 4.785, 95% CI: 2.602–8.799), FTC subtype (HR: 0.302, 95% CI: 0.090–1.022), and size ≥2 cm (2–4 cm, HR: 0.206, 95% CI: 0.114–0.375; >4 cm, HR: 0.243, 95% CI: 0.088–0.675) were significant prognostic factors for LNR. In LR/DR, age ≥55 years, *TERT*-MT, size of 2–4 cm, and positive resection margin were identified as factors associated with recurrence in the univariate regression analysis. All of these were also significant prognostic factors for cancer recurrence in the multivariate survival analysis (age ≥ 55 years, HR: 2.649, 95% CI: 1.187–5.913; *TERT*-MT, HR: 2.464, 95% CI: 0.989–6.149; size of 2–4 cm, HR: 0.142, 95% CI: 0.063–0.323; and positive resection margin, HR: 2.931, 95% CI: 1.322–6.499). In both LNR and LR/DR analyses, *TERT*-MT and a tumor size of 2–4 cm were significant risk factors for recurrence. The proportional hazards assumption was evaluated using Schoenfeld residuals, and no violation of the assumption was observed in any Cox model (TR: *P* = 0.33; LNR: *P* = 0.36; and LR/DR: *P* = 0.66).

### Impacts of *TERT* on the DFS of patients with DTC

**Figure 1:**
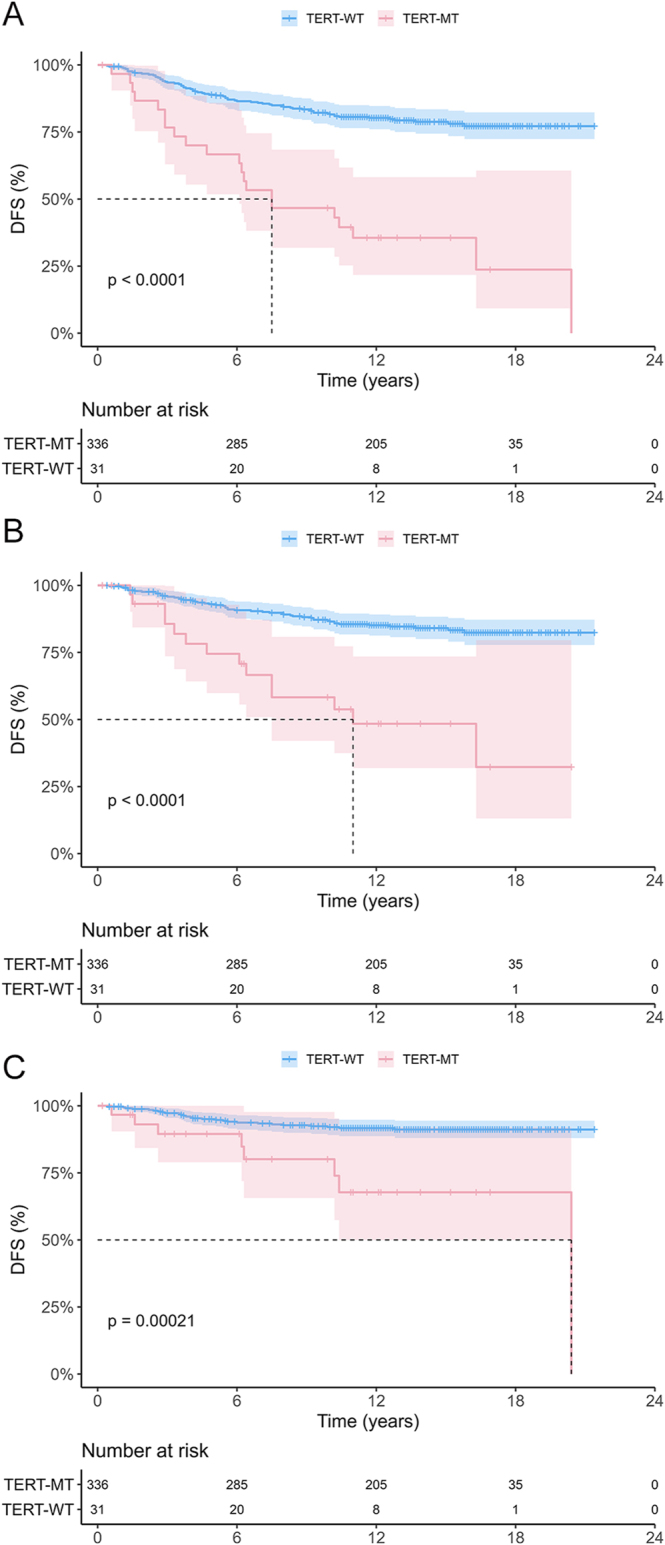
Kaplan–Meier analyses of the impact of *TERT* on the DFS of patients with DTC. Analysis results for patients with (A) TR, (B) LNR, and (C) LR/DR. *TERT, TERT* promoter mutations; DFS, disease-free survival; DTC, differentiated thyroid cancer; TR, total recurrence; LNR, lymph node recurrence; LR/DR, local soft tissue recurrence or distant recurrence; MT, mutant type; and WT, wild type. A full color version of this figure is available at https://doi.org/10.1530/ERC-25-0273.

The Kaplan–Meier analysis confirmed a significant association between *TERT*-MT and recurrence across all sites (Fig. 1). In addition, the curve for *TERT*-MT declined continuously over time compared to *TERT*-wild type (WT), indicating persistent recurrence in patients with *TERT*-MT regardless of the time elapsed.

### Impacts of *TERT* on the OS of patients with recurring DTC

**Figure 2:**
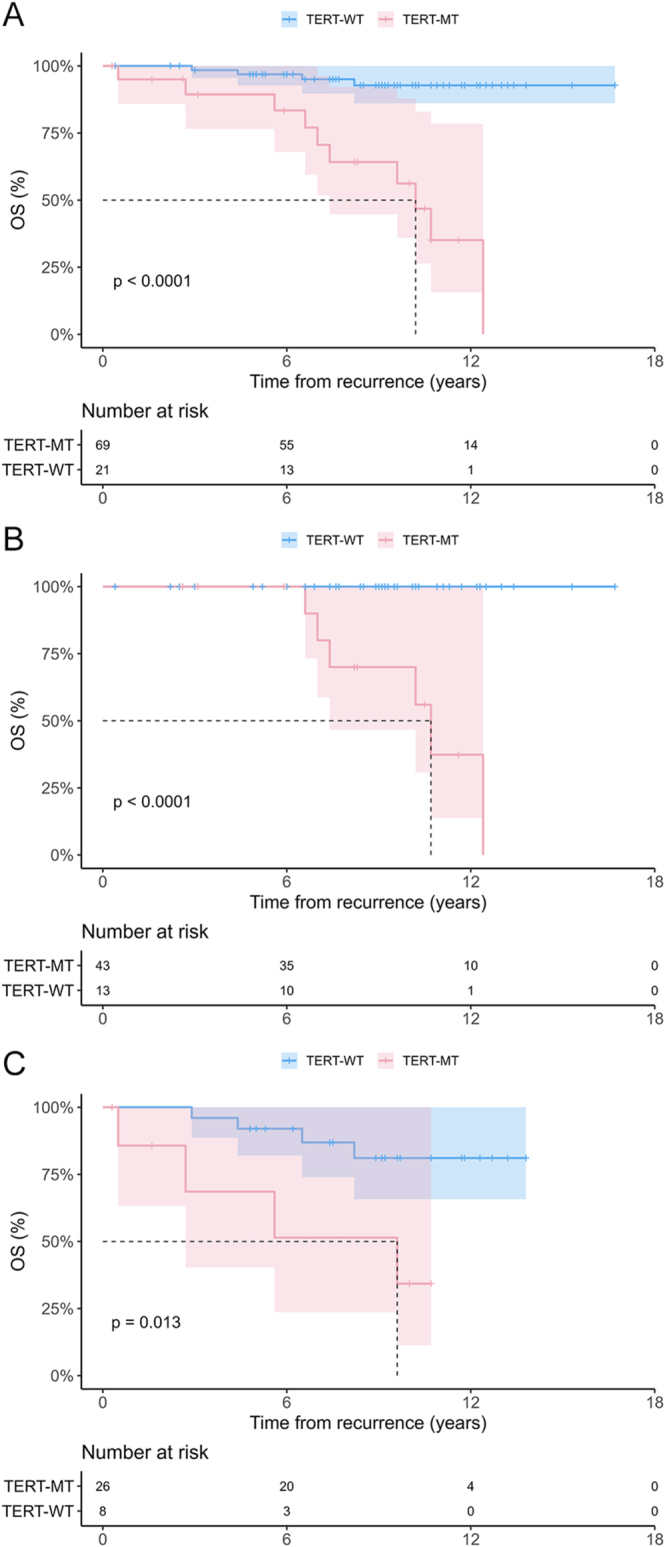
Kaplan–Meier analyses of the impact of *TERT* on the OS following recurrence of patients with DTC. Analysis results for patients with (A) TR, (B) LNR, and (C) LR/DR. *TERT, TERT* promoter mutations; OS, overall survival; DTC, differentiated thyroid cancer; TR, total recurrence; LNR, lymph node recurrence; LR/DR, local soft tissue recurrence or distant recurrence; MT, mutant type; and WT, wild type. A full color version of this figure is available at https://doi.org/10.1530/ERC-25-0273.

Upon analysis of the OS among the 91 patients who experienced recurrence (Fig. 2), *TERT*-MT was found to be significantly associated with mortality across all recurrence types. In the LNR group, no deaths occurred during the first six years of follow-up irrespective of *TERT* mutation status; however, with an extended follow-up, a significant divergence in survival emerged between the *TE* *RT*-MT and *TERT*-WT groups. In the case of *TERT*-WT, survival remained favorable even in the presence of recurrence. In the multivariate analysis, the adjusted HR showed that *TERT*-MT and sex (male) were significant risk factors for death in the TR group (Supplementary Table 2). Among the 276 patients who did not experience recurrence, no cancer-specific deaths occurred, whereas among the 91 patients who experienced recurrence, cancer-specific death occurred in 14. An additional review was conducted on the 14 patients with cancer-specific deaths (Table 3), wherein 6 experienced LNR and 8 experienced LR/DR as their first recurrence. Among the 14 patients, 10 had *TERT*-MT and 4 had *TERT*-WT.

**Table 3:** Characteristics of the patients who died.

No	Age (years)	Sex	Subtype	Histology	*TERT*	Size (cm)	Gross ETE	Initial LNM	8th TNM stage	RM	Multifocality	OP type	RAI (total dose)	Site of recurrence	Time to recurrence (years)	Cancer-specific death time (years)	Cause of death
1	68	F	PTC	Favorable	WT	3.5	N	N	1	N	N	Total	380	DR (lung)	2.2	8.7	Lung metastasis
2	54.9	M	PTC	Favorable	WT	4.0	N	N	1	N	N	Total	860	DR (lung)	9.2	17.4	Lung metastasis
3	43.9	M	PTC	Favorable	WT	7.0	N	Central	1	P	P	Total	800	DR (bone)	0.4	4.8	Bed-ridden state due to progressive bone metastasis
4	81.4	F	PTC	Favorable	MT	3.5	N	Central and lateral	2	N	P	Total	60	LNR	6.1	13.5	Local progression
5	54.2	M	PTC	Favorable	MT	1.5	N	N	1	N	P	Total	800	LNR	2.9	9.5	Multiple metastases
6	67.1	F	PTC	Favorable	MT	3.8	Major neck structures	N	3	P	P	Total	430	LNR	2.9	13.1	Multiple metastases
7	65.8	F	PTC	Favorable	MT	3.0	N	Central	2	N	N	Total	100	LNR	3.3	10.3	Local progression
8	51.4	F	PTC	Favorable	MT	3.0	N	N	1	P	P	Total	160	LR	10.2	15.8	Lung metastasis
9	39.8	M	PTC	Favorable	MT	3.0	N	N	1	N	N	Total	200	LR	0.6	1.1	Lung metastasis
10	60.9	M	PTC	Favorable	MT	10.5	Major neck structures	Central and lateral	3	N	N	Total	880	DR (lung)	1.6	11.2	Lung metastasis
11	64.8	F	PTC	Favorable	MT	2.0	N	Central and lateral	2	N	N	Total	1,210	LR	1.5	13.9	Multiple metastases
12	75.1	F	PTC	Favorable	MT	4.0	Major neck structures	Central and lateral	3	P	N	Total	150	DR	6.2	8.9	Lung metastasis
13	71.5	F	PTC	Favorable	MT	3.0	N	Central and lateral	2	N	N	Total	510	LNR	1.4	9.6	Multiple metastases presumed (last OPD: March 24, 2014; death date: August 27, 2016)
14	60.2	M	FTC	Unfavorable	WT	4.0	N	N	1	N	N	Total	900	DR (bone)	6	8.9	Bone metastasis

No, number of patients; *TERT*, telomerase reverse transcriptase; ETE, extrathyroidal extension; LNM, lymph node metastasis; TNM, tumor/node/metastasis; RM, resection margin; OP, operation; RAI, radioactive iodine therapy; PTC, papillary thyroid carcinoma; FTC, follicular thyroid carcinoma; WT, wild type; MT, mutant type; DR, distant recurrence; LNR, lymph node recurrence; LR, local soft tissue recurrence, and OPD, outpatient department.

### Interaction between age and *TERT* in relation to recurrence and survival

We also conducted a Cox regression analysis considering the interaction effect between age and *TERT* on recurrence (Supplementary Fig. 1). In the TR group, *TERT*-MT was a significant factor, whereas age ≥ 55 years was not. Since there was no interaction effect between *TERT*-MT and age (*P* = 0.967), it was confirmed that the significant impact of *TERT* on TR did not vary with age (Supplementary Fig. 1A). The interaction between age and *TERT* was absent in both the LNR (*P* = 0.591) and LR/DR (*P* = 0.349) groups (Supplementary Fig. 1B and C).

Using the same approach, we analyzed the OS of patients with recurrence while considering the interaction effect between age and *TERT* (Supplementary Fig. 2). In the TR group, *TERT* remained a significant variable, whereas age ≥ 55 years was not significant; however, there was an interaction between the two. There was a significant difference in the extent to which the HR increased depending on *TERT* status in patients <55 years compared to patients ≥55 years (age < 55 years, *P* = 0.001; age ≥ 55 years, *P* = 0.453; interaction, *P* = 0.032) (Supplementary Fig. 2A). Similarly, in the LR/DR group, there was a notable difference in the extent to which the HR increased depending on *TERT* status in patients <55 years (*P* = 0.007) and ≥55 years (*P* = 0.926; interaction, *P* = 0.027; Supplementary Fig. 2C). In the LNR group, there was no interaction between age and *TERT*; however, due to the small number of events, conducting a meaningful statistical analysis was difficult (Supplementary Fig. 2B).

## Discussion

We analyzed the effect of *TERT* on recurrence by considering the site and time of recurrence. The frequency of *TERT*-MT was significantly higher in the group with recurrence than in the group without recurrence (*P* < 0.001). There was no significant difference in the frequency of *TERT*-MT between the LNR and LR/DR groups (*P* = 1.000). Both *TERT*-MT and a size <2 cm independently served as risk factors for recurrence. *TERT*-MT acted as an unfavorable factor for the DFS, with persistent recurrence observed over time. Conversely, *TERT*-WT exhibited a gradual flattening of the curve over time in the Kaplan–Meier analysis, reaching a plateau with positive survival outcomes, even in the presence of recurrence. These findings suggest that patients with *TERT*-MT require a vigilant long-term follow-up, whereas patients with *TERT*-WT can have intensive monitoring relaxed relatively early.

Interestingly, although *TERT*-MT is generally associated with poorer mortality outcomes, no deaths occurred during the first six years after recurrence among patients with LNR, regardless of *TERT* status. Beyond this period, survival curves then began to diverge according to *TERT* status. This pattern suggests that short-term outcomes following nodal recurrence are primarily determined by the effectiveness of local control rather than by tumor genetics, whereas the adverse impact of *TERT* tends to emerge gradually during a long-term follow-up.

In this analysis, contrary to the conventional understanding, DTC < 2 cm was shown to be a more significant risk factor for recurrence (Table 2). However, it is difficult to interpret this as a poor prognostic factor due to the small sample size. This was considered a selection bias because the study targeted patients who achieved complete remission as a primary outcome after surgery. It is likely that the effect was underestimated, as cases with larger sizes and locally advanced features were excluded. As supporting evidence, the study by Kim *et al*. (8) analyzed the entire dataset from the same institution and included patients with initial distant metastasis. The study demonstrated that a larger tumor size remained a significant risk factor for mortality, consistent with conventional notions. In addition, most recurrences occurred in the LNR group (61.5%), with LNR predominantly observed in PTC (96.4%). Given that PTC was relatively smaller compared to FTC, it may partially explain why a smaller size appeared to confer a higher risk.

The recurrence rate of FTC in the <2 cm group was 22.2%, which was lower than the 67.7% recurrence rate observed for PTCs within the same size group. This finding demonstrates a trend similar to that reported by Sfreddo *et al*. (17). In their study, *RAS*-mutant indeterminate thyroid nodules with a median diameter of 1.7 cm at the time of diagnosis were subjected to active surveillance, with 81% of the nodules demonstrating stability.

In our study, there was no interaction effect between *TERT*-MT and age across all recurrence types with respect to DFS. According to Whitney Goldner (18), it is necessary to consider age alongside the ATA risk category for individuals classified as intermediate risk. Hence, it may be possible to conduct subsequent research to investigate the influence of age and *TERT* in the ATA intermediate- or high-risk groups.

Meanwhile, regarding the OS of patients with recurrence, an interaction effect between age and *TERT* was observed in both the TR group and the LR/DR group. The interaction where the mortality risk of *TERT* varied with age is similar to the findings of Heo *et al*. (19), wherein a significant interaction between age at diagnosis, *TERT* expression, and the mortality rate was confirmed using multivariable Cox regression analysis. However, other relevant factors influencing the interaction between age at diagnosis and *TERT* expression cannot be excluded and warrant further investigation.

Several studies have demonstrated the role of *TERT* in DTC recurrence. As demonstrated by Song *et al*. (11), recurrent/persistent disease was more aggressive in patients with DTC harboring *TERT*-MT than in those with no mutations. In the log-rank test, *TERT*-MT was significantly associated with an elevated risk of recurrence. Cox regression analysis indicated that the HR for recurrence associated with *TERT* remained significantly high after adjusting for the mutational status of *BRAF* and *RAS*. In their study, upon further analysis of the additional effects of coexisting mutations, such as *BRAF* or *RAS*, with *TERT* on recurrence, it was observed that the presence of *BRAF*, *RAS*, or *TERT* alone did not significantly modify the risk of recurrence; however, their coexistence heightened the risk of recurrence. Furthermore, among high-risk ATA patients or advanced TNM stage patients, *TERT* additively increased the risk of recurrence. Kim *et al*. (10) stratified patients with DTC based on *TERT* status and categories of DRS. In their study, the group with *TERT*-MT exhibited a significantly lower recurrence-free survival. Xing *et al*. (12) reported a significant association between *TERT* expression and recurrence in 507 cases of PTC using Cox regression and Kaplan–Meier analyses. However, in their study, the coexistence of *BRAF* mutations and *TERT* was significantly associated with an increased risk of PTC recurrence compared with cases where *TERT* alone were present. In a meta-analysis by Moon *et al*. (20), a comparison with a group negative for either mutation revealed that the highest risk of PTC recurrence was observed in patients with the concomitant presence of *BRAF* V600E and *TERT*. In this study, the effects of the coexistence of the two mutations over either mutation alone could not be analyzed because of the limited data.

Although this single-center retrospective study may have limitations in interpretation, it allowed for various analyses through the utilization of long-term data and acquisition of a sufficient number of recurrent events. Notably, the LNR group with *TERT*-WT demonstrated an excellent prognosis.

To our knowledge, this is the first study to longitudinally follow up on the impact of *TERT* on recurrence, while stratifying recurrence by location. A clinical implication of our study is that our findings provide a rationale for determining the timing of surgery and allocating medical resources to patients with recurrent DTC. For instance, patients with *TERT*-WT mutations exhibit markedly lower mortality rates, even in the presence of LNR. Conversely, those with *TERT*-MT or older age groups required intensive clinical surveillance, suggesting the preferential allocation of limited medical resources to these cohorts.

## Conclusion

In summary, *TERT*, which are known risk factors for recurrence in DTC, act as adverse prognostic factors across all locations of recurrence. In the case of *TERT*-MT, continuous recurrence over time necessitates a vigilant long-term follow-up. Conversely, for *TERT*-WT, the recurrence rate significantly decreased as time progressed, allowing for a relatively shorter period of close monitoring, and therapeutic outcomes are favorable even with recurrence.

## Supplementary materials







## Declaration of interest

The authors declare that there is no conflict of interest that could be perceived as prejudicing the impartiality of the work reported.

## Funding

This work did not receive any specific grants from funding agencies in the public, commercial, or non-profit sectors.

## Author contribution statement

Tae Hyuk Kim had full access to all of the data in the study and takes responsibility for the integrity of the data and the accuracy of the data analysis. Hyun Jin Ryu performed formal analysis, drafted the original manuscript, and reviewed and edited the manuscript. Ji Hyun Yoo, Da Eun Leem, Chang-Seok Ki, Jung Hee Shin, Young Lyun Oh, Jung-Han Kim, Ji Eun Jun, Kyu Jeung Ahn, Sun Wook Kim, and Jae Hoon Chung reviewed and edited the manuscript. Joonghyun Ahn performed formal analysis. Tae Hyuk Kim and Young Ik Son conceived the study and reviewed and edited the manuscript.

## Data availability

Some or all datasets generated during and/or analyzed during the current study are not publicly available but are available from the corresponding author on reasonable request.

## Ethics statement

This study was conducted according to the guidelines of the 1964 Declaration of Helsinki. This study was approved by the Institutional Review Board of the Samsung Medical Center (SMC-IRB 2016-05-053), and the requirement for written informed consent was waived because of the retrospective nature of this study.
