# Supplementary figures and images for: TERT promoter mutations and recurrence patterns in differentiated thyroid carcinoma
Source: Endocr Relat Cancer. 2026 Feb 16;33(2):e250273. doi: 10.1530/ERC-25-0273 (PMC12921681; doi:10.1530/ERC-25-0273)

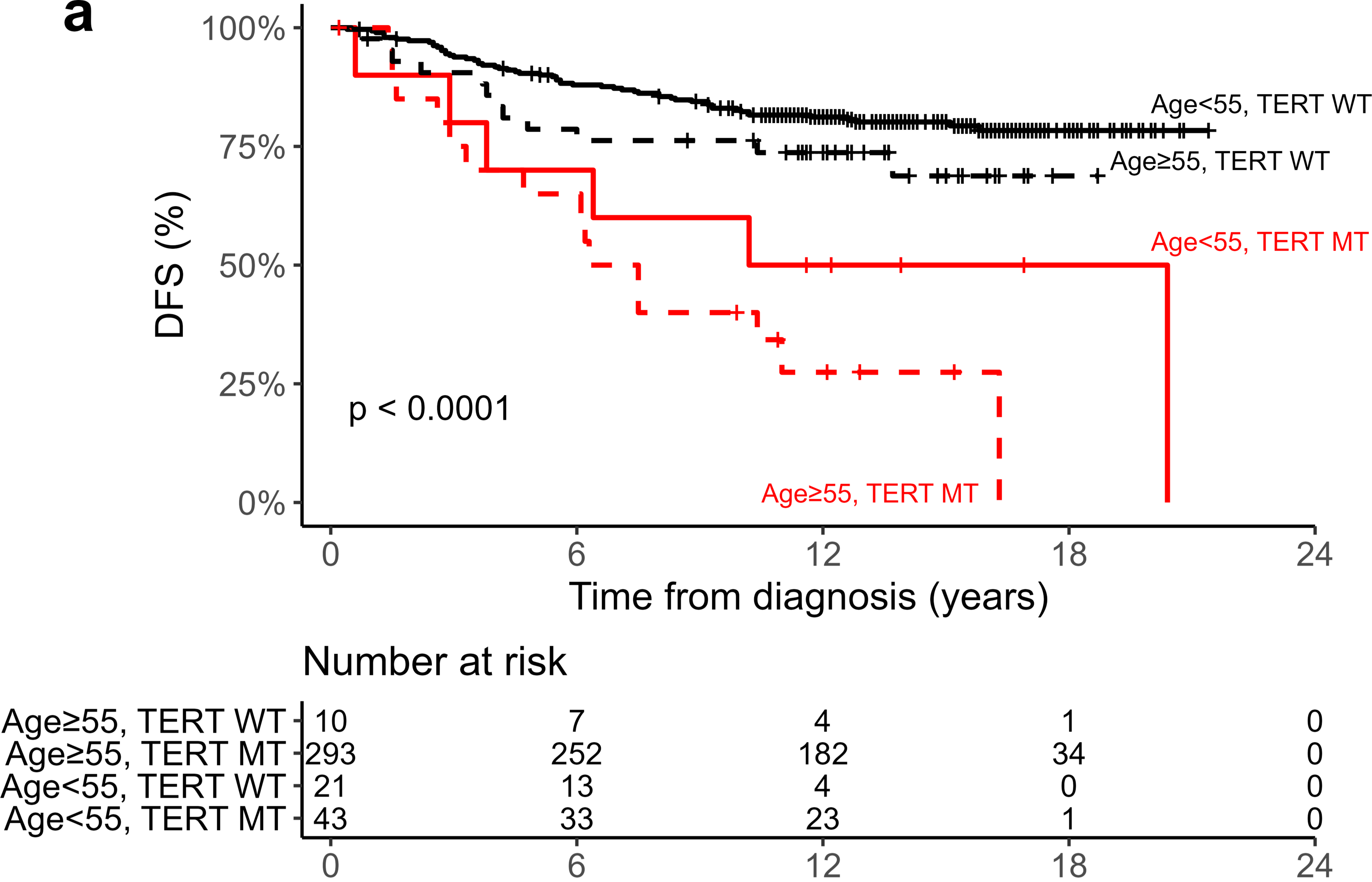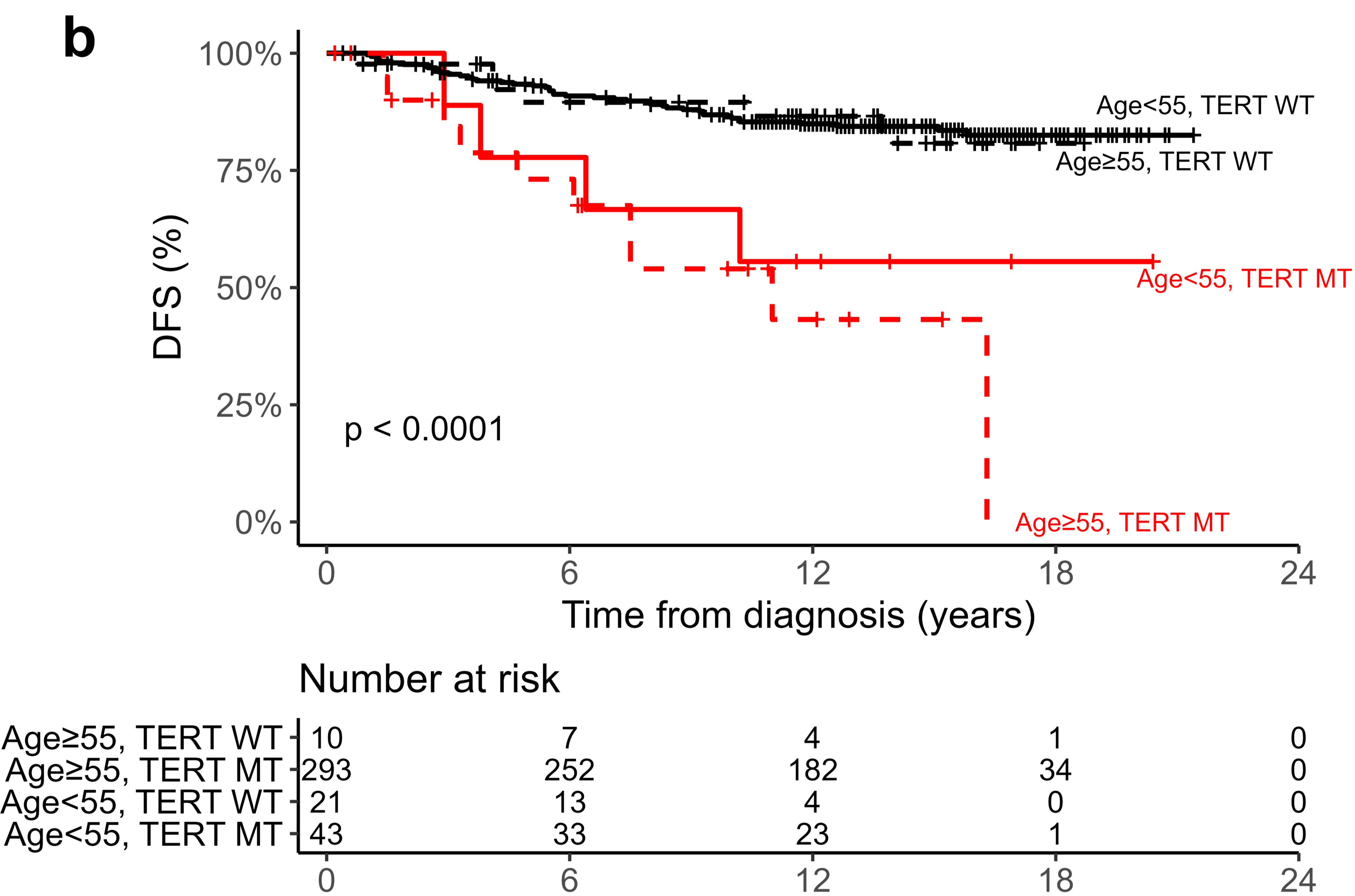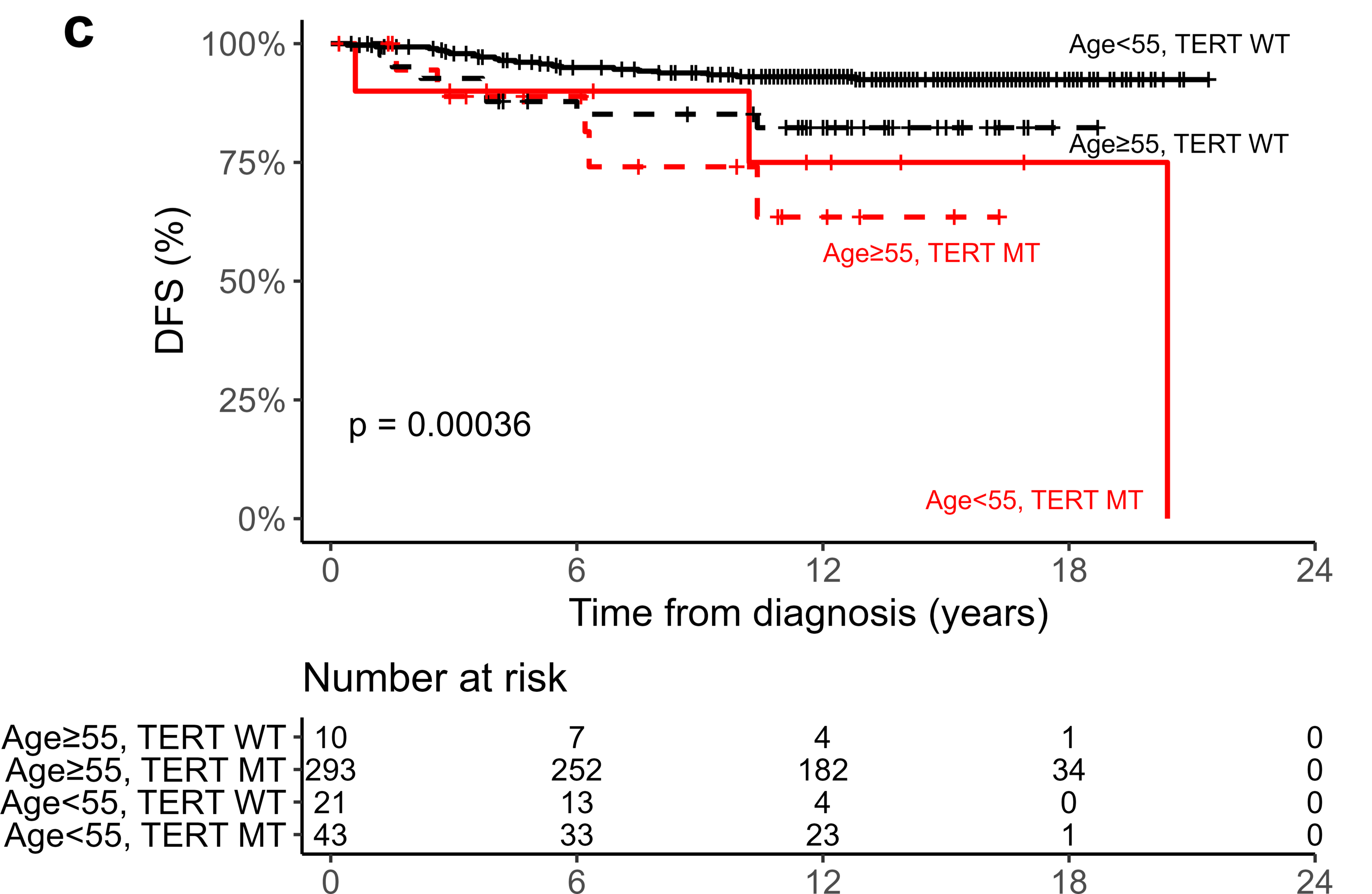

Supplement: Supplementary file 1 [file supplementary_figure_1.pdf]

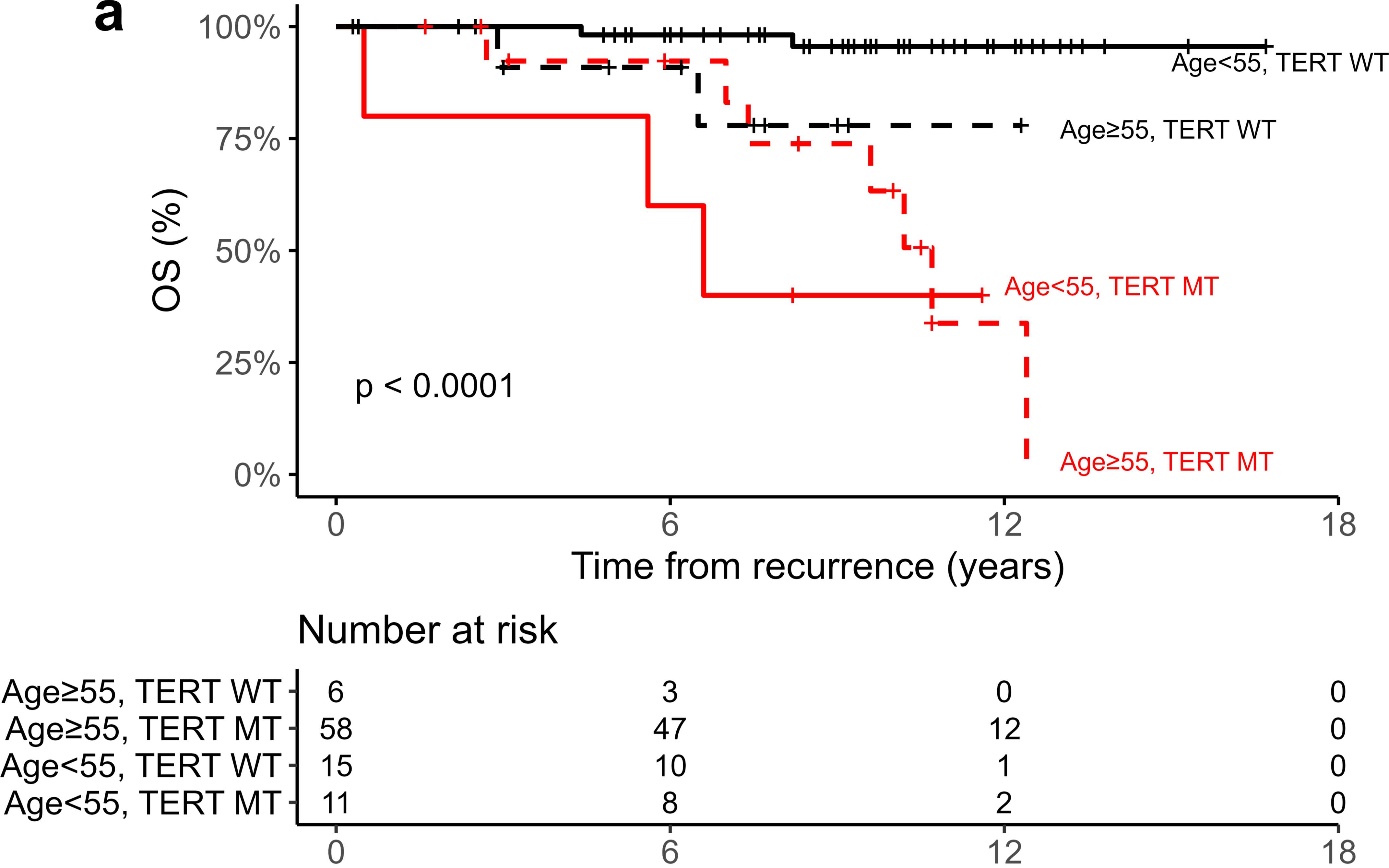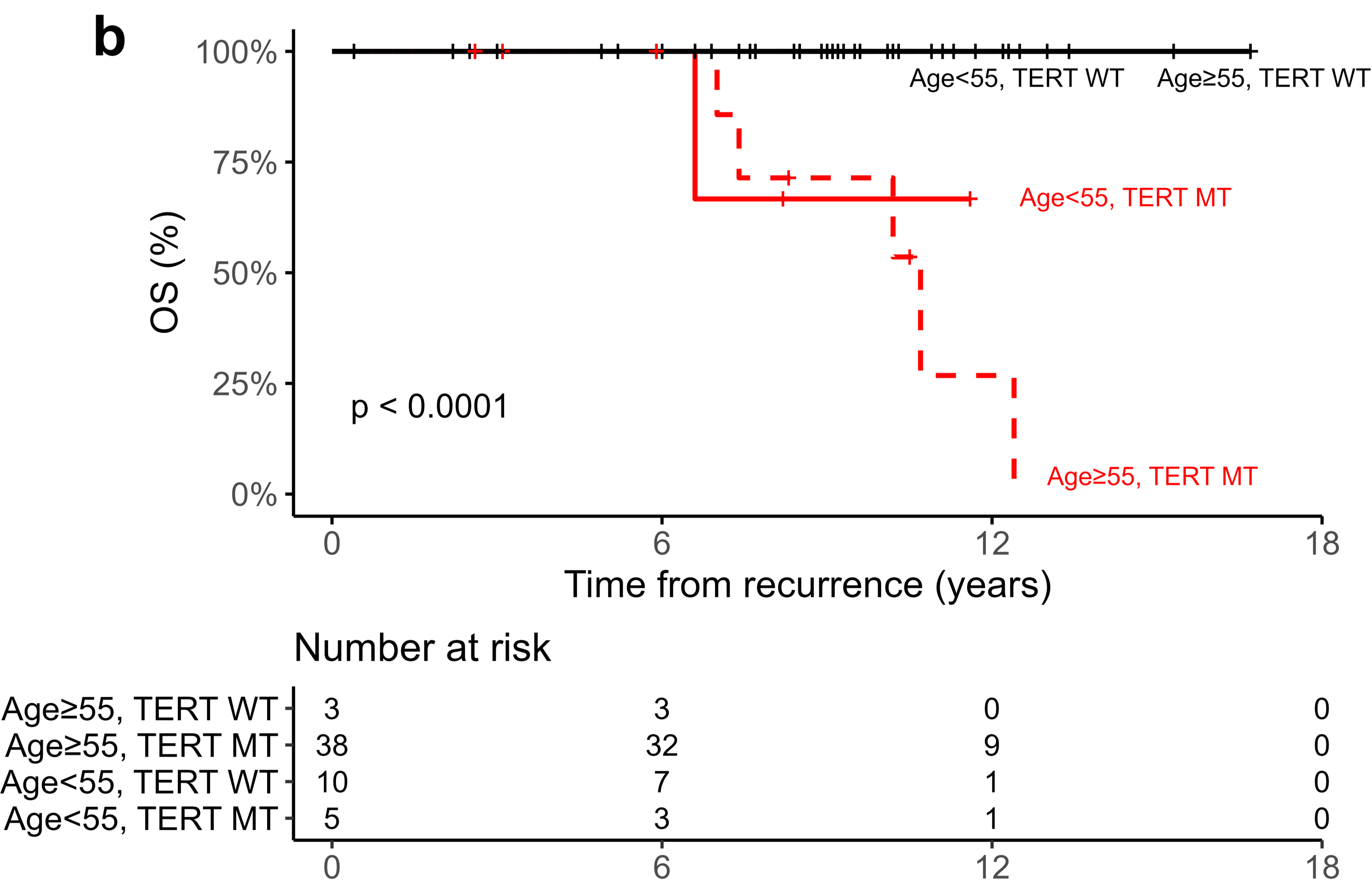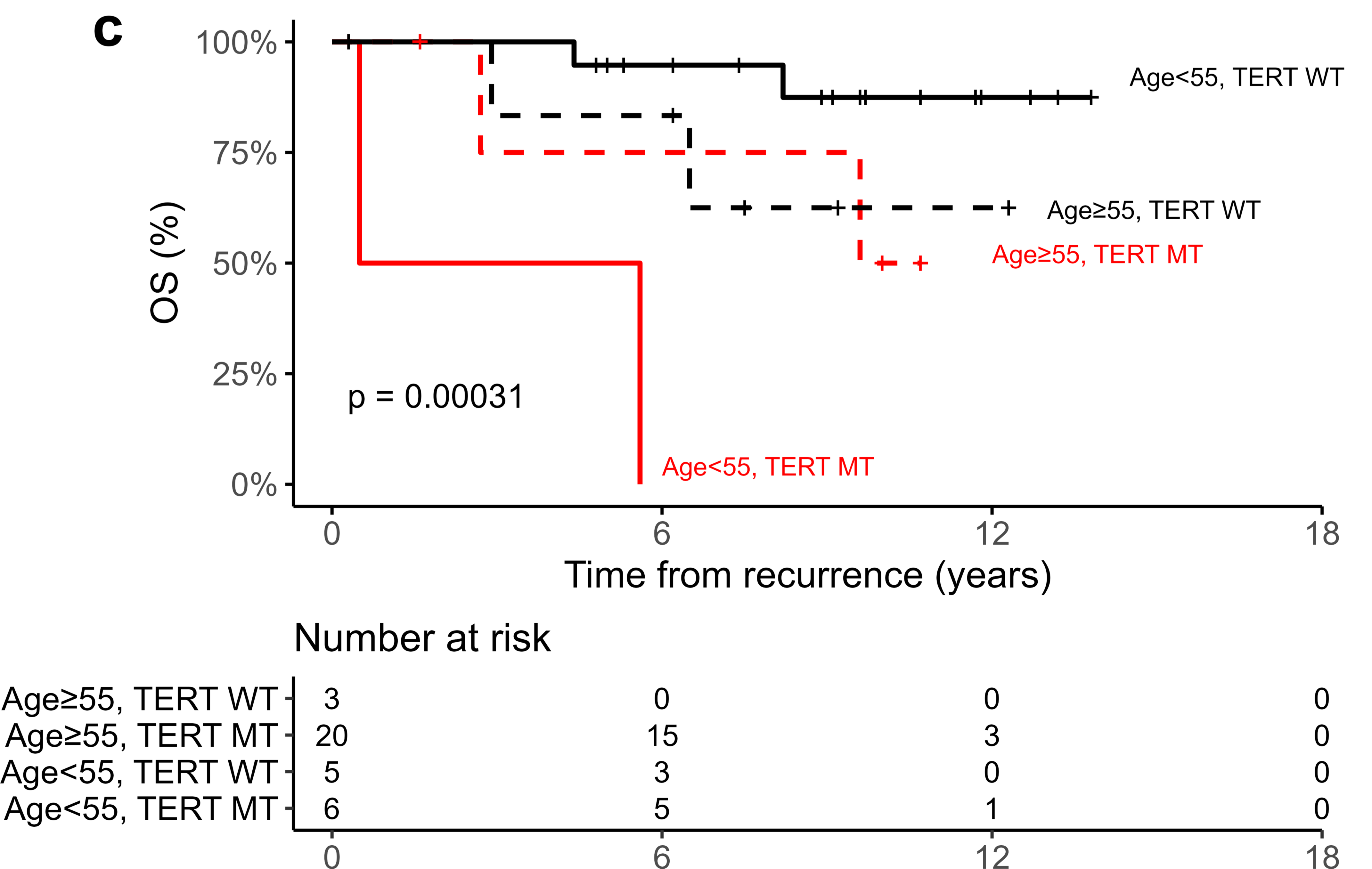

Supplement: Supplementary file 2 [file supplementary_figure_2.pdf]
