# Supplementary material for: TERT promoter mutations and recurrence patterns in differentiated thyroid carcinoma
Source: Endocr Relat Cancer. 2026 Feb 16;33(2):e250273. doi: 10.1530/ERC-25-0273 (PMC12921681; doi:10.1530/ERC-25-0273)
Supplement: Supplementary file 3 [file supplementary_tables.pdf]

**eTable 1.** Characteristics of patients according to *TERT*.

| Characteristics | Total (n=367) | TERT-WT (n=336) | TERT-MT (n=31) | *P* |
|---|---|---|---|---|
| Patient factors | | | | |
| Age | 42.3 (15.8–81.4) | 41.4 (15.8-76.1) | 59.3 (33.4-81.4) | < 0.001 |
| Age <55 | 303 (82.6) | 293 (87.2) | 10 (32.3) | |
| Female | 309 (84.2) | 283 (84.2) | 26 (83.9) | 1.000 |
| Recurrence status | | | | |
| No recurrence | 276 (75.2) | 266 (79.2) | 10 (32.2) | <0.001 |
| TR | 91 (24.8) | 70 (20.8) | 21 (67.7) | |
| LNR | 56 (61.5) | 43 (12.8) | 13 (41.9) | |
| LR/DR | 35 (38.5) | 27 (8.0) | 8 (25.8) | |
| Treatment factors | | | | |
| Type of thyroid surgery (Total) | 345 (94.0) | 317 (94.3) | 28 (90.3) | 0.232 |
| RAI | 340 (92.6) | 313 (93.2) | 27 (87.1) | 0.268 |
| Initial CND | 233 (63.5) | 215 (64.0) | 18 (58.1) | 0.560 |
| Tumor Factors | | | | |
| Subtypes (PTC) | 311 (84.7) | 284 (84.5) | 27 (87.1) | 1.000 |
| Histology (Favorable) | 341 (93.7) | 311 (93.4) | 30 (96.8) | 0.707 |
| Tumor size (cm) | 2.7 (0.4–12.0) | 2.6 (0.4-12.0) | 3.0 (1.2-10.5) | 0.524 |
| <2 cm | 40 (10.9) | 37 (11.0) | 3 (9.7) | |
| 2–4 cm | 280 (76.3) | 255 (75.9) | 25 (80.6) | |
| >4 cm | 47 (12.8) | 44 (13.1) | 3 (9.7) | |
| Multifocal tumor | 98 (26.7) | 90 (26.8) | 8 (25.8) | 1.000 |
| Gross ETE | | | | 0.002 |
| Negative | 334 (91.0) | 310 (92.3) | 24 (77.4) | |
| Only strap muscle | 21 (5.7) | 19 (5.7) | 2 (6.5) | |
| Major neck structures | 12 (3.3) | 7 (2.1) | 5 (16.1) | |
| Resection margin | 59 (16.9) | 49 (15.3) | 10 (33.3) | 0.019 |
| Central LNM | 153 (41.7) | 142 (42.3) | 11 (35.5) | 0.569 |
| Lateral LNM | 65 (17.7) | 52 (15.5) | 13 (41.9) | <0.001 |
| TNM stage | | | | <0.001 |
| Stage I | 328 (89.4) | 314 (93.5) | 14 (45.2) | |
| Stage II | 31 (8.4) | 19 (5.7) | 12 (38.7) | |
| Stage III | 8 (2.2) | 3 (0.9) | 5 (16.1) | |

The p-value was computed to compare the No recurrence group with the TR group. Values are presented as the median (minimum value–maximum value) or number (percentage).

TR, total recurrence; LNR, lymph node recurrence; LR/DR, local soft tissue recurrence or distant recurrence; RAI, radioactive iodine therapy; CND, central neck dissection; *TERT*, telomerase reverse transcriptase; MT, mutant type; PTC, papillary thyroid carcinoma; ETE, extrathyroidal extension; LNM, lymph node metastasis; TNM, tumor/node/metastasis.

**eTable 2.** Risk factors for patient mortality after recurrence.

| Variable (Reference) | TR | | | |
|---|---|---|---|---|
| | Unadjusted | | Adjusted | |
| | HR (95% CI) | *P* | HR (95% CI) | *P* |
| Age (<55) | 5.709 (1.890–17.240) | 0.002 | 3.868 (0.869–17.224) | 0.076 |
| Sex (Female) | 4.003 (1.385–11.570) | 0.010 | 7.203 (1.985–26.140) | 0.003 |
| Type of surgery (Total) | - | - | | |
| RAI (Not done) | - | - | | |
| Initial CND (Not done) | 3.475 (0.774–15. 590) | 0.104 | | |
| *TERT* (WT) | 10.710 (3.339–34.340) | <0.001 | 8.470 (2.019–35.537) | 0.004 |
| Subtype (PTC) | 2.187 (0.280–17.11) | 0.456 | | |
| Histology (Favorable) | 3.020 (0.384–23.760) | 0.294 | | |
| Tumor size | | | | |
| 1–2cm | 1.0 (reference) | - | | |
| 2–4cm | 4.552 (0.587–35.310) | 0.146 | | |
| >4cm | 3.497 (0.310–39.400) | 0.311 | | |
| Multifocal tumor (Negative) | 1.221 (0.408–3.654) | 0.721 | | |
| Gross ETE (Negative) | 2.589 (1.278–5.246) | 0.008 | | |
| Resection margin (Negative) | 1.150 (0.360–3.670) | 0.813 | | |
| Central LNM (Negative) | 1.854 (0.621–5.540) | 0.269 | | |
| Lateral LNM (Negative) | 1.076 (0.357–3.245) | 0.897 | 0.276 (0.076–1.007) | 0.051 |

CI, confidence interval; CND, central neck dissection; ETE, extrathyroidal extension; HR, hazard ratio; LNM, lymph node metastasis; PTC, papillary thyroid carcinoma; RAI, radioactive iodine therapy; *TERT*, telomerase reverse transcriptase; TR, total recurrence.
